# Potential access to primary health care: what does the National Program for Access and Quality Improvement data show?

**DOI:** 10.1590/1518-8345.1069.2672

**Published:** 2016-03-04

**Authors:** Severina Alice da Costa Uchôa, Ricardo Alexandre Arcêncio, Inês Santos Estevinho Fronteira, Ardigleusa Alves Coêlho, Claudia Santos Martiniano, Isabel Cristina Araújo Brandão, Mellina Yamamura, Renata Melo Maroto

**Affiliations:** 1 Post-doctoral fellow, Instituto de Higiene e Medicina Tropical, Universidade Nova de Lisboa, Lisboa, Portugal. Associate Professor, Departamento de Saúde Coletiva, Universidade Federal do Rio Grande do Norte, Natal, RN, Brazil. Scholarship holder from Conselho Nacional de Desenvolvimento Científico e Tecnológico (CNPq), Brazil; 2 PhD, Professor, Escola de Enfermagem de Ribeirão Preto, Universidade de São Paulo, PAHO/WHO Collaborating Centre for Nursing Research Development, Ribeirão Preto, SP, Brazil; 3 PhD, Assistant Professor, Instituto de Higiene e Medicina Tropical, Universidade Nova de Lisboa, Lisboa, Portugal; 4 PhD, Professor, Departamento de Enfermagem, Universidade Estadual da Paraíba, Campina Grande, PB, Brazil; 5 MSc, Professor, Departamento de Enfermagem, Centro Universitário FACEX, Natal, RN, Brazil; 6 Doctoral student, Escola de Enfermagem de Ribeirão Preto, Universidade de São Paulo, PAHO/WHO Collaborating Centre for Nursing Research Development, Ribeirão Preto, SP, Brazil. Assistant Professor, Escola de Enfermagem, Universidade Federal do Rio Grande do Norte, Natal, RN, Brazil. Scholarship holder from Fundação de Amparo à Pesquisa do Estado de São Paulo (FAPESP), Brazil; 7 Doctoral student, Departamento de Odontologia, Universidade Federal do Rio Grande do Norte, Natal, RN, Brazil

**Keywords:** Health Services Accessibility, Primary Health Care, Universal Coverage

## Abstract

**Objective:**

to analyze the influence of contextual indicators on the performance of municipalities regarding potential access to primary health care in Brazil and to discuss the contribution from nurses working on this access.

**Method:**

a multicenter descriptive study based on secondary data from External Evaluation of the National Program for Access and Quality Improvement in Primary Care, with the participation of 17,202 primary care teams. The chi-square test of proportions was used to verify differences between the municipalities stratified based on size of the coverage area, supply, coordination, and integration; when necessary, the chi-square test with Yates correction or Fisher's exact test were employed. For the population variable, the Kruskal-Wallis test was used.

**Results:**

the majority of participants were nurses (n=15.876; 92,3%). Statistically significant differences were observed between the municipalities in terms of territory (p=0.0000), availability (p=0.0000), coordination of care (p=0.0000), integration (p=0.0000) and supply (p=0.0000), verifying that the municipalities that make up area 6 tend to have better performance in these dimensions.

**Conclusion:**

areas 4,5 and 6 performed better in every analyzed dimension, and the nurse had a leading role in the potential to access primary health care in Brazil.

## Introduction

In 2005, members of the World Health Organization (WHO) committed to achieve the universal health coverage target provided by the Millennium Development Goals and post-2015 agenda, aiming to improve the health and welfare of the population. Universal coverage is defined as access to and appropriate use of the services according to the understanding of the health system functions; health workers who are available, motivated and qualified; access to essential medicines and health products; integrated, quality, patient-centered services; health promotion and disease control; accurate information system for adequate decision-making; and financing with protection against financial risks^(^ 1 ^)^.

There is a growing movement in this direction among the 25 richest nations and those in development, such as Brazil, Mexico and Thailand, and even in those of low-income, as Ghana, Philippines, Rwanda and Vietnam^(^ 2 ^)^. 

In Brazil, the issue of universal and equitable access has been a concern since the creation of the Unified Health System UHS (SUS) in 1988. This idea is reinforced by the National Policy of Primary Care - BANP (PNAB), in which the potential for access to comprehensive care management through multidisciplinary, interdisciplinary team work is emphasized^(^ 3 ^)^.

However, access has been strongly marked by social inequalities, with disadvantaged populations in vulnerable situations with an impact on the health status of these groups, causing more iatrogenic situations, poorer quality services and continued, more severe suffering with some health conditions, including preventable and premature deaths. Thus, new forms of system organization, with real universal coverage has been envisioned to achieve equity and integrality of actions^(^ 4 ^)^. Another challenge is shortage in the distribution, composition and competence of human resources, especially physicians, nurses and midwives^(^ 5 ^)^. In response to the most critical component, physicians, incentive programs were adopted to supply and qualify these professional, through the Enhancement Program of Primary Care, and by importing foreign physicians with the More Medical Doctors Program^(^ 6 ^)^.

A significant advance towards the access to health care services with quality and better working conditions occurred with the implementation of the first cycle of the National Program for Access and Quality Improvement in Primary Care (PMAQ-AB)^(^ 7 ^)^. The program is organized in four phases: voluntary participation of municipal managers; contracting by each Primary Care Team (PCT) of performance indicators for monitoring; development of self-assessment, institutional support and continuing education; external evaluation and re-contracting, starting a new quality cycle. In the external evaluation, seven Higher Educational Institutions (IES) investigated throughout the country, *in loco*, the structure of the Basic Health Units (BHU) (census) and the working process of the contracted Primary Care Teams (PCT). 

The complexity of the universal coverage paradigm has elicited theoretical studies in recent years^(^ 8 ^)^ on principles and repercussions in the Brazilian scenario, and some empirical studies about APS^(^ 9 ^)^; use of services^(^ 10 ^)^; medications^(^ 11 ^)^ and educational practices^(^ 12 ^)^.

Despite the contributions on the subject, national studies that evaluate the relationship between contexts and the centrality of professionals in the work teams, focusing on access and equity, remain scarce. The aim of this article is, to analyze the influence of contextual indicators on the performance of municipalities, with regard to potential access to APS in Brazil, based on external evaluation of the PMAQ-AB and to discuss the contribution of the work of nursing.

### Study design

This was a cross-sectional cohort study, using national data from the Bank of Evaluators of the External PMAQ.

### Research scenario

In 2012, SUS had 36,361 Basic Health Units (BHU) and 33,404 Family Health Teams (FHT) with coverage in 5,297 municipalities. The adherence to PMAQ occurred with 17,202 Primary Care Teams (PCT). Among these, 16,566 FHT and 636 non- FHT were distributed in 3,944 (70.8%) of the total municipalities, in 14,111 Basic Health Units (BHUs)^(^ 7 ^)^.

### Population and sample

The study population included professionals linked to the primary care team and qualified in PMAQ^(^ 7 ^)^, namely physicians, nurses, and dentists. In each team, only one sampling unit was selected for the study.

### Measurement instruments and data sources 

The questionnaires with closed-ended questions were provided in*tablets*, administered by interviewers who had the same training, under supervision. Next, they were sent online to the Ministry of Health system, accessed and validated by the IES, based on a consistency analysis protocol and validation of the data collected through the*soft* Validator's *online,* PMAQ-AB. The characteristics of respondents and four (4) dimensions of the Module II questionnaire - Interview with professional of Primary Care Team and Document Checking of the Health Unit External Evaluation of the first cycle of the PMAQ-AB, were included here for data analysis^(7).^ The dimensions that were representative of the potential levels of access according to the authors' judgment were chosen and are described in the analysis plan.

### Classification of municipalities according to the context variables

The municipalities listed in the study are classified into six strata, considering the *per capita* Gross Domestic Product (GDP), the percentage of the population with health insurance, the percentage of the population on the *Bolsa Família* (Family Grant) program, the percentage of the population in extreme poverty, and the population density.

The composition of the extracts considered for each municipality were: the lowest score among the percentage of the population with *Bolsa Família*program, and the percentage of the population in extreme poverty: area 1 - Municipalities with scores lower than 4.82 and a population of up to 10,000 inhabitants; area 2 - Municipalities with scores lower than 4.82 and a population of up to 20 thousand inhabitants; area 3 - municipalities with scores lower than 4.82 and a population of up to 50 thousand inhabitants; area 4 - Municipalities with scores between 4.82 and 5.4, and population of up to 100 thousand inhabitants; area 5 - Municipalities with scores between 5.4 and 5.85, and population of up to 500 thousand inhabitants; and municipalities with a score lower than 5.4, and population between 100 and 500 thousand inhabitants; and area 6 - Municipalities with population over 500,000 inhabitants, or a score less than 5.85^(^ 7 ^)^.

Variables under consideration to evaluate potential access:

The variables considered for evaluating potential access are described in Table 2. The table shows the dimension, characteristic and nature of the variables that are included.

**Table 1 -:** Characteristics of study sample, PMAQ Project, Brazil (2012)

**Variables**	**PMAQ Areas**							
	**1**	**2**	**3**	**4**	**5**	**6**		
Professional category *n ( %)*								
	Physician	72 (0.42)	59 (0.34)	52 (0.30)	91 (0.53)	143 (0.83)	576 (3.35)	
	Nurse	2.058 (11.96)	2.179 (12.67)	2.425 (14.10)	3.119 (18. 13)	2.615 (15.20)	3.480 (20.23)	
	Dentist	35 (0.20)	35 (0.20)	50 (0.29)	56 (0.33)	56 (0.33)	101 (0.59)	
Years of work/experience *n (%)*								
	Less than 1 year	546 (3.17)	693 (4.03)	801 (4.66)	995 (5.78)	830 (4.83)	875 (5.09)	
	Between 1-3 years	867 (5.04)	966 (5.62)	1.068 (6.21)	1.384 (8.05)	1.133 (6.59)	1.598 (9.29)	
	Greater than three years	743 (4.32)	608 (3.53)	652 (3.79)	881 (5.12)	843 (4.90)	1.673 (9.73)	
	Don´t know/ no response	9 (0.05)	6 (0.03)	6 (0.03)	6 (0.03)	8 (0.05)	11 (0.06)	
Type of team *n (%)*								
	Family Health Teams with oral health	1.832 (10.66)	1.798 (10.45)	2.041 (11.86)	2.464 (14.32)	1.767 (10.27)	2.173 (12.63)	
	Family Health Teams without oral health	261 (1.52)	398 (2.31)	423 (2.46)	720 (4.19)	942 (5.48)	1.824 (10.60)	
	Primary care team with oral health	59 (0.34)	57 (0.33)	45 (0.26)	59 (0.34)	57 (0.33)	51 (0.30	
	Primary care teams without oral health	7 (0.04)	9 (0.05)	11 (0.06)	15 (0.09)	43 (0.25)	39 (0.23)	
	Others	4 (0.02)	6 (0.03)	4 (0.02)	7 (0.04)	3 (0.02)	66 (0.38)	
	Do not Know/No response	2 (0.01)	5 (0.03)	3 (0.02)	1 (0.01)	2 (0.01)	4 (0.02)	
Minimum number of physicians in the primary care staff of BHU (n= 16643)								
	Median	1	1	1	1	1	1	
	Minimum and Maximum value	0.00 - 4.00	0.00 - 4.00	0.00 - 4.00	0.00 - 11.00	0.00 - 11.00	0.00 - 6.00	
Minimum number of nurses in the primary care staff (n=16643)								
	Median	1	1	1	1	1	1	
	Minimum and maximum value	0.00 - 4.00	0.00 - 4.00	0.00 - 4.00	0.00 - 4.00	0.00 - 4.00	0.00 - 4.00	
Minimum number of dentists in the primary care staff (n=16643)								
	Median	1	1	1	1	1	1	
	Minimum and maximum value	0.00 - 6.00	0.00 - 4.00	0.00 - 3.00	0.00 - 6.00	0.00 - 6.00	0.00 - 4.00	
Minimum number of nursing technicians in the primary care staff (n=16643)								
	Median	1	1	1	1	1	1	
	Minimum and maximum value	0.00 - 13.00	0.00 - 10.00	0.00 - 10.00	0.00 - 8.00	0.00 - 20.00	0.00 - 11.00	
Minimum number of nursing assistants in the primary care staff (n=16643)								
	Median	0	0	0	0	0	1	
	Minimum and maximum value	0.00 - 9.00	0.00 - 8.00	0.00 - 8.00	0.00 - 8.00	0.00 - 6.00	0.00 - 20.00	
Minimum number of dental technicians in the primary care staff (n=16643)								
	Median	0	0	0	0	0	0	
	Minimum and maximum value	0.00 - 8.00	0.00 - 8.00	0.00 - 8.00	0.00 - 2.00	0.00 - 3.00	0.00 - 8.00	
Minimum number of dental assistants in the primary care staff (n=16643)								
	Median	1	1	1	1	1	0	
	Minimum and maximum value	0.00 - 6.00	0.00 - 7.00	0.00 - 8.00	0.00 - 9.00	0.00 - 8.00	0.00 - 10.00	
Minimum number of community health workers in the primary care staff (n=16643)								
	Median	6	6	7	6	6	5	
	Minimum and maximum value	0.00 - 19.00	0.00 - 50.00	0.00 - 42.00	0.00 - 50.00	0.00 - 56.00	0.00 - 32.00	
Allowing the patient to choose team by which he wants to be treated *n (%)*								
	Yes	219 (1.27)	191 (1.11)	180 (1.05)	161 (0.94)	127 (0.74)	303 (1.76)	
	No	286 (1.66)	309 (1.80)	303 (1.76)	411 (2.39)	442 (2.57)	1.059 (6.16)	
	Not applicable	454 (2.64)	539 (3.13)	516 (3.00)	671 (3.90)	355 (2.06)	196 (1.14)	
	Don´t know/No response	1.206 (7.01)	1.234 (7.17)	1.528 (8.88)	2.023(11.76)	1.890 (10.99)	2.599 (15.11)	
								

**Table 2:** - Performance of municipalities on patient access according to the areas, Brazil, 2012

**Dimension**	**Variables**	**PMAQ areas**							
		**1**	**2**	**3**	**4**	**5**	**6**	**p value**	
		**n (%)**	**n (%)**	**n (%)**	**n (%)**	**n (%)**	**n (%)**		
Personal qualification	Complementary education (n=17.202)								
		Yes	1.708 (9.93)	1.795 (10.43)	2.050 (11.92)	2.694 (15.66)	2.460 (14.30)	3.642 (21.17)	0.000*
		No	457 (2.66)	478 (2.78)	477 (2.77)	572 (3.33)	354 (2.06)	515 (2.99)	
	Career development programs (n=16.936)								
		Yes	253 (1.49)	159 (0.94)	246 (1.46)	574 (3.39)	581 (3.43)	1.810 (10.69)	0.000*
		No	1.877 (11.08)	2.069 (12.22)	2.245 (13.26)	2.647 (15.63)	2.194 (12.95)	2.279 (13.46)	
	There are continuing education activities involving primary care professionals (n=17.113)								
		Yes	1.432 (8.37)	1.596 (9.33)	1.878 (10.97)	2.601 (15.20)	2.481 (14.50)	3.969 (23.19)	0.000*
		No	720 (4.21)	658 (3.85)	630 (3.68)	650 (3.80)	325 (1.90)	173 (1.01)	
Coverage area	How many people for whom the team is responsible								
		Mean	2165	2273	2527	3266	2814	4157	0.0001^†^
	Risk and vulnerability criteria were considered for defining people for whom the team is responsible (n=15.691)								
		Yes	1.024 (6.53)	1.141 (7.27)	1.323 (8.43)	1.705 (10.87)	1.423 (9.07)	2.648 (16.88)	0.000*
		No	951 (6.06)	877 (5.59)	937 (5.97)	1.265 (8.06)	1.115 (7.11)	1.282 (8.17)	
	There is definition of team coverage area (n=17.150)								
		Yes	2.086 (12.16)	2.197 (12.81)	2.456 (14.32)	3.190 (18.60)	2.763 (16.11)	4.113 (23.98)	0.000*
		No	68 (0.40)	60 (0.35)	63 (0.37)	71 (0.41)	43 (0.25)	40 (0.23)	
	There is a population uncovered by primary care surrounding the team's coverage area (n=17.092)								
		Yes	369 (2.16)	534 (3.12)	888 (5.20)	1.083 (6.34)	1.391 (8.14)	1.513 (8.85)	0.000*
		No	1.783 (10.43)	1.724 (10.09)	1.618 (9.47)	2.170 (12.70)	1.406 (8.23)	2.613 (15.29)	
	How often people from outside the team's coverage area are served by this team (n=16.855)								
		Every day of the week	900 (5.34)	828 (4.91)	1.001 (5.94)	1.247 (7.40)	1.255 (7.45)	2.152(12.77)	0.000*
		Some days of the week	966 (5.73)	1.135 (6.73)	1.201 (7.13)	1.502 (8.91)	1.222 (7.25)	1.673 (9.93)	
		Any day of the week	248 (1.47)	243 (1.44)	266 (1.58)	451 (2.68)	287 (1.70)	178 (1.65)	
Availability	Patients who spontaneously arrive and have their needs heard and assessed (n=17.140)								
		Yes	2.121 (12.37)	2.202 (12.85)	2.442 (14.25)	3.180 (18.55)	2.689 (15.69)	4.078 (23.79)	0.000*
		No	38 (0.22)	59 (0.34)	80 (0.47)	83 (0.48)	108 (0.63)	60 (0.35)	
	The team performs risk and vulnerability assessment in the intake of patients (n=13.739)								
		Yes	1.265 (9.21)	1.385 (10.08)	1.645 (11.97)	2.286 (16.64)	2.050 (14.92)	3.442 (25.05)	0.0066*
		No	192 (1.40)	221 (1.61)	248 (1.81)	324 (2.36)	236 (1.72)	445 (3.24)	
	The schedule is organized to conduct home visitation (n=13.951)								
		Yes	1.418 (10.16)	1.628 (11.67)	1.865 (13.37)	2.391 (17.14)	2.253 (16.15)	3.697 (26.50)	0.000*
		No	134 (0.96)	115 (0.82)	114 (0.82)	149 (1.07)	104 (0.75)	83 (0.590)	
Coordination of care	Keep a record of high risk patients referred to other points of care (n=17.104)								
		Yes	826 b(4.83)	818 (4.78)	1.104 (6.45)	1.474 (8.62)	1.353 (7.91)	2.385 (13.94)	0.000*
		No	1.310 (7.66)	1.439 (8.41)	1.405 (8.21)	1.785 (10.44)	1.449 (8.47)	1.756 (10.27)	
	There is a document proving (n=								
		Yes	605 (7.60)	638 (8.02)	913 (11.47)	1.206 (15.15)	1.132 (14.22)	1.978 (24.85)	0.000*
		No	221 (2.78)	180 (2.26)	191 (2.40)	268 (3.37)	221 (2.78)	407 (5.11)	
	There are protocols that guide the prioritization of cases needing referral (n=17.037)								
		Yes	581 (3.41)	613 (3.60)	807 (4.74)	1.213 (7.12)	1.228 (7.21)	2.907 (17.06)	0.000†
		No	1.558 (9.14)	1.636 (9.60)	1.685 (9.89)	2.036 (11.95)	1.567 (9.20)	1.206 (7.08)	
Integration	There is a regulation center (n=17.201)								
		Yes	1.880 (10.93)	2.006 (11.66)	2.239 (13.02)	2.907 (16.90)	2.540 (14.77)	4.027 (23.41)	0.000*
		No	284 (1.65)	267 (1.55)	288 (1.67)	359 (2.09)	274 (1.59)	130 (0.76)	
	There is a referral form for patients moving to other points of care (n=17.201)								
		Yes	1.752 (10.19)	1.828 (10.63)	2.138 (12.43)	2.970 (17.27)	2.615 (15.20)	4.055 (23.57)	0.0000*
		No	412 (2.40)	445 (2.59)	389 (2.26)	296 (1.72)	199 (1.16)	102 (0.59)	
Supply	Receive enough basic medicines from pharmacy to serve its population (n=17.161)								
		Yes	1.459 (8.50)	1.490 (8.68)	1.722 (10.03)	2.210 (12.88)	1.830 (10.66)	2.898 (16.89)	0.0000*
		No	378 (2.20)	457 (2.66)	614 (3.58)	644 (3.75)	718 (4.18)	2.077 (6.28)	
		Do not receive	316 (1.84)	320 (1.86)	187 (1.09)	406 (2.37)	263 (1.53)	172 (1.00)	
	Offers service of complementary and integrative practices for patients of the area (n=17.199)								
		Yes	235 (1.37)	230 (1.34)	305 (1.77)	381 (2.22)	512 (2.98)	1.546 (8.99)	0.0000*
		No	1.929 (11.22)	2.042 (11.87)	2.222 (12.92)	2.885 (16.77)	2.301 (13.38)	2.611 (15.18)	
	Conducts home visits (n=17.199)								
		Yes	2.146 (12.48)	2.262 (13.15)	2.521 (14.66)	3.253 (18.91)	2.802 (16.29)	4.148 (24.12)	0.0075*
		No	18 (0.10)	10 (0.06)	6 (0.03)	13 (0.08)	11 (0.06)	9 (0.05)	
	The families in the coverage area are visited at intervals according to risk and vulnerability assessment? (n=17.132)								
		Yes	1.963 (11.46)	2.069 (12.08)	2.345 (13.69)	2.997 (15.30)	2.621 (15.30)	3.986 (23.27)	0.0000*
		No	183 (1,07)	193 (1,13)	176 (1,03)	256 (1,49)	181 (1,06)	162 (0,95)	

* p value statistically significant (p<0.05) † Kruskal-Wallis test

### Plan of analysis

Initially, the descriptive analysis of the characteristics area of the municipalities', professional category, and median number of professionals per team was calculated. 

Regarding the performance of municipalities in terms of access, four dimensions of the PMAQ instrument were measured: coverage area, supplies, customer coordination, and integration.

The variables were dichotomized into yes and no. Thereafter, the sum of the responses for each item was calculated, dividing this number by the total sample. To verify differences between the municipalities in relation to the size of potential access, the chi-square test of proportions was used. The chi-square test with Yates or Fisher's exact test correction was applied when necessary. For the population variable, the Kruskal-Wallis test was used to verify differences in relation to the median inhabitants monitored by areas.

After the analysis of the performance of the municipalities within the areas, in relation to access, multivariate statistics by multiple correspondence analyses (MCA) was used, given that the instrument variables were categorical.

 The MCA implementation was based on the steps of Spencer^(^ 13 ^)^ and Mingoti^(^ 14 ^)^, in which the tabulation of responses generated a matrix, with rows corresponding to the participating health professionals, and the columns corresponding to the variables. Subsequently, the matrix turned into a complete disjunctive table (CDT). In the table, the columns represent characteristics of the variables, in which the intersection of Row I with Column J is the *xij*, which is 0 or 1, indicating that the area either has or does not have the characteristic.

The perceptual map was formed by this technique, which is a visual representation of the variables in two or more dimensions. Each variable has a spatial position in the perceptual map, variables perceived as similar or associated are allocated to proximal points on the map, while those not perceived as similar are represented as distal points. The proximity indicates the correspondence between the categories represented in rows and columns of the table.

 The component row or column influences the construction of the axes through its inertia, in relation to the center of gravity. The inertia means the variance of the data set ^(^ 13 ^)^. From the MCA it was possible to extract the most representative dimensions in terms of inertia, which in the study corresponded to the first two. Its contribution to inertia was considered a criterion for selection of the variables.

## Results

Table 1 shows characteristics of the sample of 17,202 teams recruited for the study, according to the PMAQ area. The majority of participants were nurses (n =;%), and many of them had less than three years of experience after completing their education.

Among the models of care, in all areas, there was a predominance of the Family Health Strategy (FHS) without oral health. In general, there is a median of one (1) physician, nurse, nursing technicians, and dentist per team. All modalities of care investigated showed that most of the teams did not provide the patient with the opportunity to choose a desired unit for treatment and follow up.

In Table 2, the performance of municipalities in terms of patient access is verified, considering the area established in PMAQ.

Statistically significant differences were identified between the municipalities of area 1, 2 and 3 with area 4, 5 and 6, and the professionals of the last areas had more qualifications (p=0.0000).

Regarding the career plan, no statistically significant difference (p = 0.0000) was observed, and the municipalities of area 4, 5 and 6 had better indicators; lowest values were found in areas 1, 2 and 3. Also, these areas showed statistically significant differences associated with their training policy and continuing education (p=0.0000).

According to Table 2, statistically significant differences in t erms of population coverage were observed, in which area 5 and 6 monitored a median number of people with access well above that of areas 1, 2 and 3. Also, statistically significant differences were present between the municipalities in terms of coverage area (p=0.0000), availability (p=0.0000), coordination of care (p=0.0000), integration (p=0.0000) and supply (p=0.0000), verifying that the municipalities that form area 6 tend to have better performance in these dimensions.

When compared by professional category (Table 3), a statistically significant difference is again identified, in which a higher proportion of both physicians as well as dentists tend to refer to more positive aspects of their units than nurses.

**Table 3:** - Performance of primary care for patient access to the health system according professional category, Brazil, 2012

**Variables**	**Professional Category**	**P value**											
	**Physician**	**Nurse**	**Dentist**										
	**Yes**	**%**	**No**	**%**	**Yes**	**%**	**No**	**%**	**Yes**	**%**	**No**	**%**	
Complementary education n=17202	800	4.6	193	1.1	13285	77.2	2591	15.1	264	1.5	69	0.4	0.0046*
Career development programs n=17113	303	1.8	670	4.0	3224	19.0	12412	73.3	98	0.6	229	1.4	0.0000*
Continuing education activities n=17113	853	5.0	132	0.8	12850	75.1	2951	17.2	254	1.5	73	0.4	0.0000*
All patients have their needs heard and assessed n=17047	956	5.6	25	0.15	15362	90.1	380	2.2	309	1.8	15	0.1	0.0384*
The team performs risk assessment during the intake n=13730	777	5.6	95	0.7	11066	80.6	1538	11.2	223	1.6	31	0.2	0.5189
Schedule is organized for home visitation n=11473	743	6.5	27	0.2	10013	87.3	480	4.2	201	1.8	9	0.1	0.3815
High risk patients are registered when referred n=13658	488	3.6	378	2.8	6261	45.9	6284	46.0	136	1.0	111	0.8	0.0004*
Form to register the patient referral n= 6885	377	5.5	111	1.6	5159	75.0	1102	16.0	107	1.6	29	0.4	0.0105*
Protocols that guide the prioritization of cases for referral n=13606	533	3.9	329	2.4	5797	42.6	6704	49.3	129	1.0	114	0.8	0.0000*
Regulation center for referral n=17047	905	5.3	76	0.4	14274	83.7	1468	8.6	292	1.7	32	0.2	0.2347
Forms for referral of patients n=17047	915	5.4	66	0.4	14029	82.3	1713	10.1	294	1.7	30	0.2	0.0001*
Sufficient medicines in primary care to meet population needs n=17015	606	3.6	373	2.1	10721	63.0	4992	30	205	1.2	118	0.7	0.0000*
Offering integrative and complementary practices n=17045	273	1.6	707	4.2	2865	16.8	12877	75.6	46	0.3	277	1.6	0.0000*
The team performs home visitation n=17045	977	5.7	4	0.02	15690	92.1	52	0.31	320	1.9	3	0.02	0.1846
The families of coverage area are frequently visited	927	5.5	50	0.3	14636	86.2	1054	6.2	289	1.7	31	0.2	0.0142*

* p <0,05

The proportion of nurses who tends to identify weaknesses in relation to the organization of services is much greater than other professionals.

In complementary education, for example, whereas there is one "No" for each 4 "Yes" assigned by physicians in this item, and almost one "No" for each three "Yes" assigned by dentists, among nurses this proportion was almost five, which was statistically significant (p = 0.0046). Career development programs was also another point on which this difference was very significant (p = 0.0000), where again, the proportion of nurses who reported the absence of or lack of participation in was much higher than other categories.

When a comparative analysis of the APS related to the models of care was conducted, the FHT with or without oral health predominated. Statistically significant differences were identified in career development program variables, where the proportion of professionals linked to the FHT, which has career development programs, was much smaller than the professionals integrated in other models of care (p=0.0000). Similarly, a statistically significant association regarding continuing education activities (p=0.0000) was observed, records of the documentation of cases referred for other services (p=0.0462), protocols to guide professionals for referrals to other services (p=0.0000) and use of complementary practices (p=0.0000). A significant difference was observed in the home visits, where the FHT presented a higher proportion of visits compared to the other two forms of attention (p=0.0000). 

**Table 4:** - Performance of primary care for access to the patient according to the model of care, Brazil, 2012

**Activities**	**Model of care**		**P value**										
	**FHT (with or without oral health)**	**Team AB**	**Other model**										
	**Yes**	**%**	**No**	**%**	**yes**	**%**	**No**	**%**	**Yes**	**%**	**No**	**%**	
Complementary education - V23 n= 17185	13883	80.8	2760	16.1	383	2.2	69	0.4	75	0.4	15	0.1	0.3059
Career development programs n=16923 v24	3516	21.0	12876	76.1	99	0.6	344	2.0	7	0.1	81	0.5	0.0000*
Continuing education activities = 17100 v25	13487	78.9	3074	18.0	283	2.2	66	0.4	80	0.5	10	0.1	0.0000*
All patients have their needs heard and assessed n=16987 v31	16055	94.6	397	2.3	422	2.5	15	0.1	85	0.5	3	0.0	0.1754
The team performs risk assessment during the intake n= 13723 v32	11710	85.3	1626	11.8	283	2.1	33	0.2	66	0.5	5	0.1	0.3987
Schedule is organized for home visitation n= 11473 v33	10678	93.1	486	4.2	236	2.1	22	1.32	43	0.4	8	0.1	0.3815
High risk patients are registered when referred n= 13658 v34	6685	50.0	6588	48.2	167	1.2	147	1.1	33	0.2	38	0.3	0.1323
Form to register the patient referral n= 6885 v35	5483	79.6	1202	17.5	136	2.0	31	0.5	24	0.4	9	0.1	0.0462*
Protocols that guide the prioritization of cases for referral n= 13606 v36	6289	46.2	6930	51.0	145	1.1	171	1.3	25	0.2	46	0.3	0.0000*
Regulation center for referral n= 17047 v37	12232	90.0	997	7.3	283	2.1	24	0.18	67	0.5	3	0.1	0.6982
Forms for referral of patients n= 17047	14782	86.7	1728	10.1	370	2.2	77	0.5	86	0.5	4	0.1	0.0000
V39 Has / receives medicines n= 17045	11146	59.5	5333	31.3	316	1.9	130	0.8	70	0.4	20	0.1	0.0286
V40 Offering integrative/ complementary practices n= 17045	3082	18.1	13426	78.8	93	0.6	354	2.1	9	0.1	81	0.5	0.0000*
V41 Team performs home visitation n = 17045	16462	96.6	46	0.3	437	2.6	10	0.1	88	0.5	2	0.1	0.0000*
V42 Families of coverage area are frequently visited n= 16987	15363	90.4	1099	6.5	404	2.4	33	0.2	85	0.5	3	0.1	0.1092

*p< 0,05

**Figure 1:**
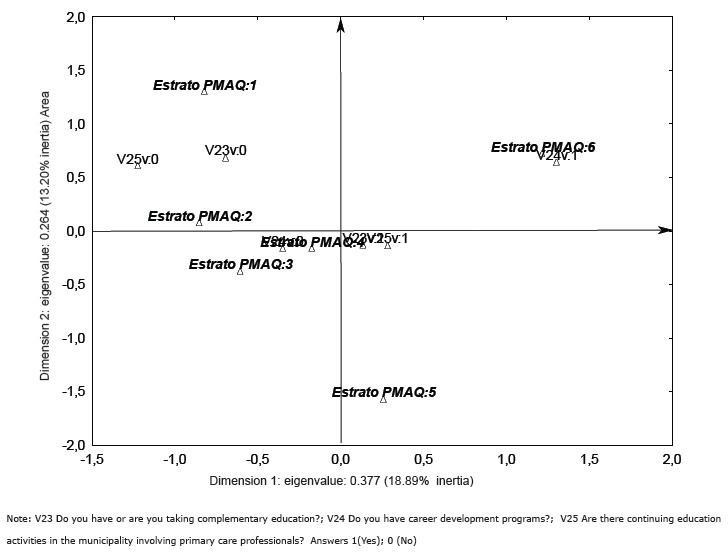
- Qualification for professionals working in the context of primary health care, according to the area of PMAQ, Brazil (2012)

The Multiple Correspondence Analysis enabled the creation of the perceptual map shown in Figure 1, which demonstrates that the map can be divided into quadrants; on the right side, quadrants are plotted municipalities that showed better indicators in terms of qualification than those on the left.

This figure demonstrate that the municipalities that comprise areas 5 and 6 present better indicators with regard to the training of their health professionals; the municipalities that are concentrated closer to the center have regular values. Thus they had some satisfactory indicators and others that were unsatisfactory, and municipalities of areas 1 and 2 had less satisfactory indicators for this item.

**Figure 2:**
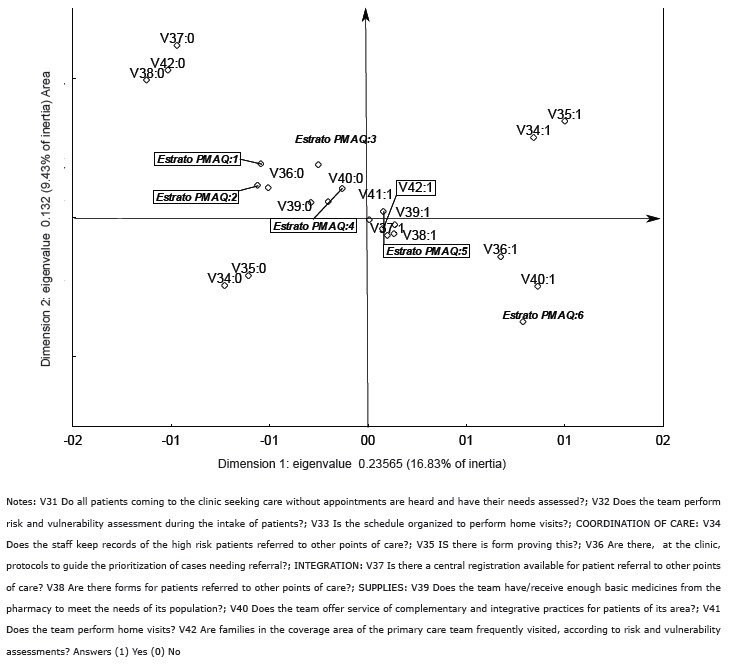
- Performance of municipalities for access to primary care according to the area defined by PMAQ, Brazil (2012)

Figure 2 expresses the performance of municipalities in terms of availability, coordination of care, integration and supply using a perceptual map. On the right side of the map, the municipalities that showed better indicators are represented, and on the left side are those with poorer indicators.

Considering this evaluation with all of these attributes, the single area with satisfactory indicators across all of these dimensions was area 6; the municipalities of area 4 and 5 showed median values, with satisfactory indicators in some of those and unsatisfactory in others; however, the municipalities of area 5 were better than area 4; the municipalities of area 1, 2 and 3 did not achieve satisfactory results in these dimensions.

## Discussion

The prevailing participation of nurses as respondent in all area reveals their involvement with this level of assistance. In this sense, they are potentially able to cooperate with the UHC coverage by their role in all health care levels, and their particular desire to contribute to the achievement of the goal. The organization of nurses in international networks has been recognized by the PAHO/WHO, with an emphasis on achieving UHC and access to health care for the entire population ^(5)^.

In the assessment of the contextual or socioeconomic indicators and health, and the influence of professional qualification and territorial process in APS, areas 4, 5 and 6 showed better performance in all analyzed dimensions.

The best performance of the professional qualification in the present study, in areas 4, 5 and 6, was also observed in a study conducted in large cities, where more than half of physicians and nurses had participated in some training process in the prior 30 days^(^ 15 ^)^. 

Although a statistically significant difference was found between the areas with respect to career plan, all areas showed a weak performance in this item, which can be explained by the way in which professionals are recruitment. A study, conducted in Minas Gerais, showed that 75% of municipal health secretaries use temporary contracts for provision of services by professionals with higher education^(^ 16 ^)^.

This study highlights significant findings on the existence of continuing education actions. Continuing professional development is important, using information and communication technologies that facilitate the qualification of these professionals for the job. Such strategies also contribute to improving the problem solving within the FHU, and promote communication between specialists and generalists^(^ 17 ^)^.

With regard to coverage areas in Brazil, currently, the population coverage estimated by the APS teams becomes important as an universal indicator of success with the guidelines and goals of SUS^(^ 18 ^)^. It is necessary to note that, although the average number of persons under the responsibility of the team is within the recommendation of the Ministry of Health^(^ 3 ^)^, this number is considered high, if we consider that, in Brazil, the teams are responsible for a large number of activities^(^ 19 ^)^. 

To enable access to the population that is not covered by primary care, teams comply with the principle of universality, but also tend to undergo activity overloads, considering that more and more frequently the APS/FHT have new responsibilities delegated to them, and face responsibilities for diseases, priority groups, problems or specific situations^(^ 20 ^)^. A similar situation is seen in the UK and Europe, where professionals also develop a wide range of tasks, which include, among others: prevention activities, acute care/curative activities, treatment for patients with chronic conditions, and emergency treatment. These professionals are responsible for a roster of almost 2,250 people^(^ 21 ^)^.

Regarding availability, the unscheduled demand by patients to have their needs met and evaluated occurred in all areas, with better performance in areas 4, 5 and 6. These findings differ from those found by Giovanela, Fausto and Fidelis, which showed barriers to spontaneous demand and non-priority groups. Home visits are on the professional schedules in all areas of the municipalities. Similarly, this activity was observed as a routine of physicians and nurses in four large cities^(^ 22 ^)^. When comparing the models of care, there was a predominance of home visits being conducted by the FHT, a similar result to that found in a study with southern and northeastern cities^(^ 10 ^)^.

In the coordination of care, despite the significant differences between the areas, all areas presented unsatisfactory performance regarding the registration of referrals to other points of care, featuring a referral process without accountability and relationship with the patient.

In the integration of care, the existence of a central registration is present in the municipalities of the area analyzed, predominantly in 4, 5 and 6. Similar results were noted by physicians and nurses of the FHT that recognized the existence of a central registration for appointments and exams^(^ 23 ^)^.

With regard to the provision of health actions and services, there was a statistical significance in all aspects evaluated. The availability of medicines in the basic pharmacy to meet the population was observed in municipalities of all areas. In some cities of the country, this distribution is more related to priority groups^(^ 15 ^)^. It is remarkable to note the low supply of complementary and integrative practices for patients of the area, which may be linked to the fact that this type of care integrates a specialized service network, such as acupuncture offered in Porto Alegre^(^ 24 ^)^.

In the work process of the APS teams, the nurse takes on several assignments, among them: planning, individual and collective care, management, and systematic assessment of developed actions (PNAB. 20123), which may justify the tendency of nurses to negatively evaluate the actions of the organization. In the daily nursing work of the FHT units, difficulties occur, mainly related to lack of training for implementation of actions^(^ 25 ^)^. 

Regarding the contribution of nurses to universal access, the study showed that the majority were nurses, which shows in a way the involvement of this category of professional with the APS. The nurse has a more focused training for this area, with well-aligned curricula to the SUS social policy, with content in anthropology and sociology, health management, leadership and community sanitation practices, making her more sensitive to innovations in the context of the APS, and more motivated to promote change.

One important issue is that most nurses eventually assume leadership in the teams, strategically, and taking the forefront of primary care as a new mode of social production in health. The low pay of these professionals in the private sector makes many find the SUS to provide a chance for stability, which is very positive in terms of securing professionals in that category. One challenge is the establishment of a new model that values their core competence and recognizes their autonomy in prescribing and care. The hegemonic model with centrality in medical practice tends to push them out of this process.

## Limitations

The study was not conducted in all the Brazilian municipalities, and only in those in which the teams voluntarily qualified for the PMAQ; thus, the results should be interpreted with caution because they do not retain the ability to be generalized. There is the possibility of selection bias, as not all staff members were included; only one staff member was chosen, and this was voluntary. Additionally, the study has design limitations, as it is a cross-sectional design, and is guided by interviews of professional. There was no monitoring of the teams for a period of time, or triangulation of data obtained from interviews with others, such as observation, records or statements of patients, which would increase the accuracy of the findings. However, it is important to note that the PMAQ is the first evaluation of this scope and methodological homogeneity and, despite the limitations, the findings contribute in the advancement of knowledge regarding APS-enhanced access, its critic nodes and also a situational diagnosis of which municipalities have advanced more in terms of universal coverage systems and those which have not.

## Conclusion

The study showed that there is a relationship between access and socioeconomic conditions: as the area of the municipalities increases, the access to services tends to be better. However, within a context of social inequalities and iniquities, weaknesses are perceived that jeopardize the organization of health activities in the municipalities regarding the availability, care coordination, integration, and supply, particularly in the municipalities grouped in areas 1 to 3. Given the involvement of the nurse with the organization of health care, this professional has contributed to the potential access of APS in Brazil.
